# Endosinotarsal device exerts a better postoperative correction in Meary’s angle than exosinotarsal screw from a meta-analysis in pediatric flatfoot

**DOI:** 10.1038/s41598-020-70545-6

**Published:** 2020-08-11

**Authors:** Chiun-Hua Hsieh, Chia-Che Lee, Tzu-Hao Tseng, Kuan-Wen Wu, Jia-Feng Chang, Ting-Ming Wang

**Affiliations:** 1 National Taiwan University Hospital, No. 7, Zhongshan S. Rd., Zhongzheng Dist., Taipei, Taiwan; 2 Department of Orthopedic Surgery, No.7, Chung Shan S. Rd., Taipei, 100 Taiwan, ROC; 3 Division of Nephrology, Department of Internal Medicine, Shuang Ho Hospital, Taipei Medical University, New Taipei City, 235 Taiwan, ROC; 4 Division of Nephrology, Department of Internal Medicine, En Chu Kong Hospital, New Taipei City, 237 Taiwan, ROC

**Keywords:** Bone, Musculoskeletal abnormalities, Bone

## Abstract

For pediatric flexible flatfoot, the subtalar extra-articular screw arthroereisis (SESA) and endosinotarsal device are the most popular techniques in current practice. Nevertheless, scarce literature is available comparing the outcomes between these two techniques. Thus, we aimed to provide a meta-analysis for the radiographic and clinical outcomes, respectively. A systemic search for correction of pediatric flexible flatfoot using subtalar arthroereisis was conducted mainly in Pubmed and Scopus, and the search was completed on 31 Dec., 2019. The standardized mean differences (SMD) of postoperative versus preoperative calcaneal pitch and Meary’s angle were defined as the primary outcomes, whereas the preoperative versus posteoperative AOFAS (American Orthopaedic Foot and Ankle Society) as the secondary outcome. The meta-analysis included 12 comparative studies comprising 2063 feet in total. The quantitative analysis showed a marked improvement in Meary’s angle of endosinotarsal cone implant group (SMD: 4.298; 95% CI 2.706–5.889) than exosinotarsal screw group (SMD: 1.264; 95% CI 0.650–1.877). But no significant difference was noted between both groups in calcaneal pitch and AOFAS. The exosinotarsal screw and endosinotarsal device are both effective arthroereisis implant for pediatric flexible flatfoot. While considering the correction of Meary’s angle, the endosinotarsal device is better than exosinotarsal screw.

## Introduction

For pediatric flexible flatfoot, the subtalar extra-articular screw arthroereisis (SESA) and endosinotarsal device are the most popular techniques in current practice. Nevertheless, we found that there were limited studies to compare the outcomes between these two techniques. Traditionally, exosinotarsal screw is thought as inexpensive, while endosinotarsal implant is thought to preserve more soft tissue. However, none of the study has provided the difference between the two procedures^1^. Although we further went through the literature focusing on outcomes of exosinotarsal screw and endosinotarsal device for subtalar arthroereisis procedure, the comparison study is still lacking^2^. Furthermore, there was no multi-center, multi-surgeon study to compare these two techniques in the previous research. Thus, we aimed to provide a meta-analysis for radiographic and clinical outcomes, respectively.

## Material and methods

### Search strategy and inclusion criteria

Pubmed, Scopus, Cochrane Collaboration Central Register of Controlled Clinical Trials, Cochrane Systemic Review, and ClinicalTrials.gov were searched for studies concerning the use of extraosseous subtalar arthroereisis for pediatric flexible flatfoot from the earliest record (September 1974, till 31 Dec., 2019). The bibliography of included trials and related review articles were manually reviewed for relevant reference. Literature not written in English, not available in full text, adult patients, focus on gait analysis, review articles, patients with neuromuscular diseases, case reports, or techniques except exosinotarsal subtalar screw in calcaneus or endosinotarsal cone-shaped implant were excluded. We investigated studies using endosinotarsal tunnel subtalar arthroereisis for pediatric flexible flatfoot treatment.

The search strategy comprised the following keywords combined with subtalar arthroereisis: flexible flatfoot and pes planus. Regarding the types of included studies, we enrolled randomized controlled trials (RCTs), comparative experimental trials, or single-armed follow up studies. We excluded case series and case reports. The target population comprised pediatric patients who suffered from painful flexible flatfoot.

### Data extraction and quality assessment

Two reviewers examined all of the retrieved articles and extracted data using a predetermined form. We recorded the first author, year, sample size, implant choice, combination of soft tissue procedure, radiographic outcome, and clinical outcome. The methodological quality of enrolled studies was independently evaluated by two reviewers using Jadad scoring for the RCTs and the Newcastle–Ottawa Quality Assessment Scale for the comparative experimental trials.

The Newcastle–Ottawa Quality Assessment Scale contains 9 items in 3 categories: participant selection (4 items), comparability (4 items), and exposure (3 items)^3^ A study can be scored a maximum of one point for each item in the Selection and Exposure domains and a maximum of 2 points for each item in the Comparability domain. Between-reviewer discrepancies were solved through discussions under the supervision of the corresponding author.

### Data synthesis and analysis

The standardized mean differences (SMDs) of calcaneal pitch and Meary’s angle between the SESA group and endosinotarsal device group comprised the primary outcomes. Data extracted from the radiographic outcome parameters were evaluated during outpatient department follow up postoperatively. A negative SMD value indicated receiving arthroereisis surgery was a worse option. AOFAS (American Orthopaedic Foot and Ankle Society) score was also retracted, and a negative SMD value of AOFAS indicated receiving arthroereisis surgery was a worse option.

A random effects model was employed to pool individual SMDs. All analyses were performed using CMA software V3. Between-trial heterogeneity was determined by using I2 tests; values > 50% were regarded as considerable heterogeneity^4^.

Funnel plots and Egger’s test were used to examine potential publication bias^5^. Statistical significance was defined as *p*-values < 0.05.

## Results

**Figure 1:**
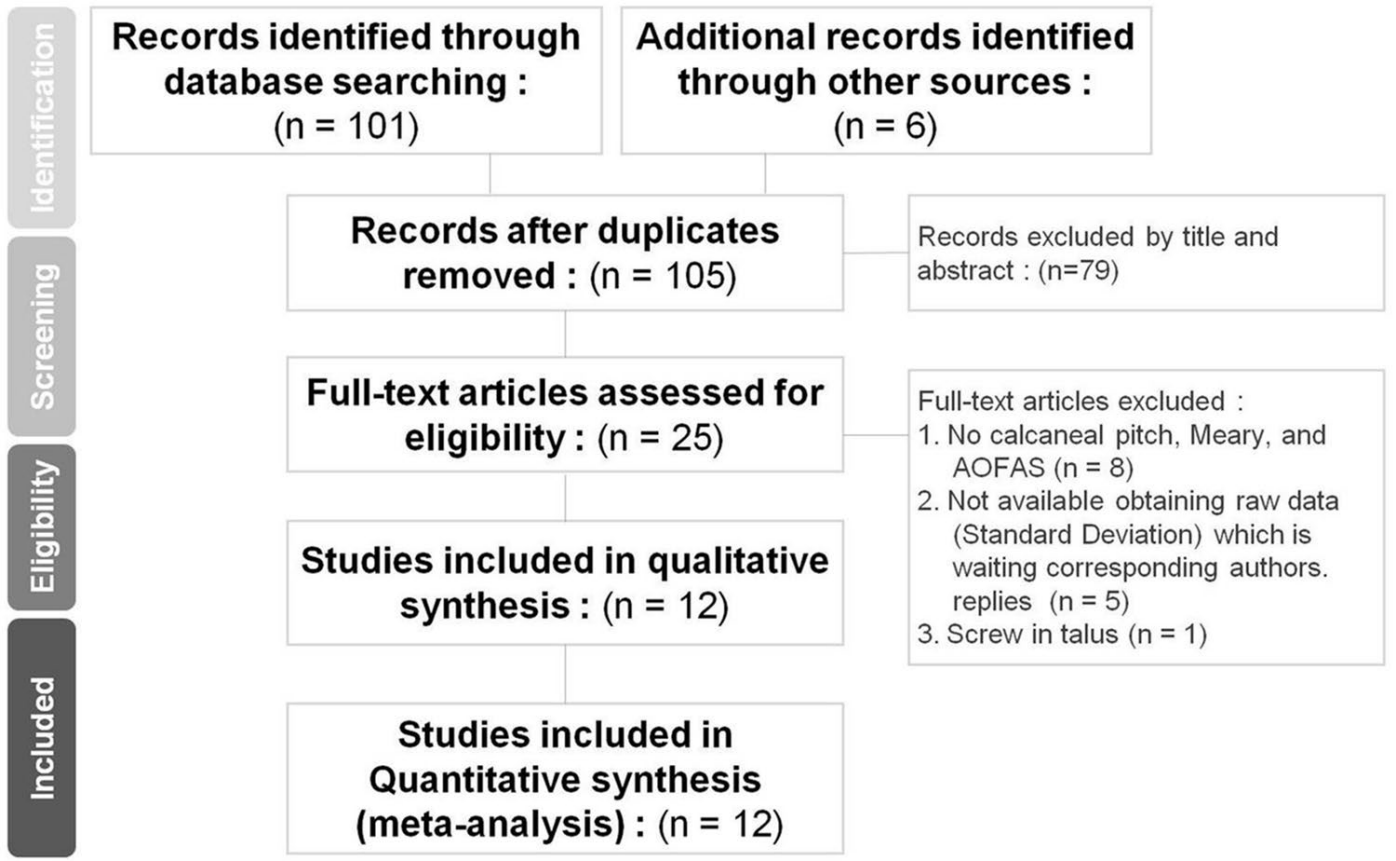
Systematic reviews and flow diagram for the searching and identification of included studies.

We retrieved 101 non-duplicated citations for reviewing their titles and abstracts and other 4 articles using manual extraction. We finally included 12 articles for meticulous evaluation after eliminating references violating inclusion criteria (Fig. 1).

We divided the study groups from Memeo in JFAS into endosinotarsal group and exosinotarsal group^1^. Thus, we have 12 groups in total.

After writing emails to corresponding authors, there had been no response until 31 Dec., 2019. We excluded 1 study for screw inserted in talus^6^; 7 studies for lack of calcaneal pitch angle, Meary’s angle, and AOFAS^7–13^ and 6 studies for lack of standard deviation or raw data^14–19^.

In terms of the patient population, 6 studies performed with endosinotarsal cone shaped implant^20–23^, 5 studies performed with exosinotarsal screw^24–29^, while one study performed with both surgeries were divided into two groups^1^.

The final quantitative analysis included 2063 feet. Ten studies were one-armed retrospective studies and the other one was two-armed retrospective study^1^. Patient age was 4 to 17 years in endosinotarsal cone-shaped implant group and 4 to 16 years in exosinotarsal group. The pediatric flexible flatfoot was the only diagnosis. Patient characteristics, study methodology, and quality assessment of included trials were listed in Table 1. The collected data of each article were listed in Table 2.

**Table 1:** Summary of patient characteristics, study methodology, implant and quality assessment of trials for pediatric flexible flatfoot diagnosis retrieving from each article.

Implant	Author, year, journal	Study Design	Arthroereisis implant	Age at op	Male/female (Feet)	Viladot classification	Implant (± soft tissue)	Quality Assessment
		**RCT vs. quasi-E**	**Extraosseous vs intraosseous**					**Newcastle–Ottawa quality assessment scale**
Endosino tarsal device	Bernasconi A (2019) Orthop Traumatol Surg Res	Quasi-E	Endosinotarsal device	10.5 ± 1.6	45M/17F	Not mention	Expanding non-reabsorbable Giannini implant	7
Endosino tarsal device	Megremis P (2019) J Foot Ankle Surg	Quasi-E	Endosinotarsal device	10.71 ± 1.58	10M/4F (20:8)	Not mention	Intergra + Hoke	7
Endosino tarsal device	Hsieh CH (2019) JCM	Quasi-E	Endosinotarsal device	9 ± 2	59M/43F (118:86)	3–4	Bioarch + Vulpius	7
Endosino tarsal device group solely	Memeo A (2019) J Foot Ankle Surg (endosinotarsal group)	Quasi-E	(Endosinotarsal device group)	12.8 ± 4	61M/39F (122:78)	3–4	Endosinotarsal cone + two level Achilles lengthen	7
Endosino tarsal device	Cao L (2017) Orthop Surg	Quasi-E	Endosinotarsal device	12.1 ± 5	12M/8F (not mention)	Not mention	Kalix II	6
Endosino tarsal device	Jay RM (2013) Foot Ankle Spec	Quasi-E	Endosinotarsal device	10.6 ± 6	13M/7F (not mention)	Not mention	Endosinotarsal cone + gastrocnemius recession	5
Intra-osseous screw	Kubo H (2019) J Orthop Sci	Quasi-E	Intra-osseous screw	10 ± 5	Not mention	Not mention	Exosinotarsal screw + Baumann, Strayer, or Z lengthening of Achilles tendon	7
Exosino tarsal screw group solely	Memeo A (2019) J Foot Ankle Surg (exosinotarsal group)	Quasi-E	(Exosinotarsal screw group)	13.6 ± 3	50M/51F (100:102)	Not mention	Exosinotarsal screw + percutaneous Achilles lengthen	7
Intraossoeus screw	Giannini S (2017) J Foot Ankle Surg	Quasi-E	Intraossoeus screw	6 ± 2	31M/1 F (62:26)	2–4	Cannulated RSB + percutaneous Achilles lengthening	6
Intraosseous screw	De Pellegrin M (2014) J Child Orthop	Quasi-E	Intraosseous screw	11.5 ± 1.81	267M/218F (not mention)	Not mention	Metallic screw	7
Intraosseous screw	Jerosch J (2009) Foot Ankle Surg	Quasi-E	Intraosseous screw	11.9 ± 3	13M/5F (not mention)	Not mention	Metallic screw + Baumann	6
Intraosseous screw	V. Pavone (2018) J Child Orthop	Quasi-E	Intraosseous screw	12.7 ± 3	38M/30F (76:60)	Not mention	Calcaneal stop screw (Synthes)	7
Intraosseous screw	Pavone V (2013) J Foot Ankle Surg	Quasi-E	Intraosseous screw	11 ± 3	157M/85F (not mention)	Not mention	Calcaneal stop screw (Synthes) + Achilles tendon lengthening	7

**Table 2:** Summary of corrective figures of calcaneal pitch, Meary's angle and AOFAS under pre- and post-op status retrieving from each article.

Implant	Author, year, journal	Preop Calcaneal pitch			Postop Calcaneal pitch			Preop AP Meary’s angle			Postop AP Meary’s angle			Preop lateral Meary’s angle			Postop lateral Meary’s angle			Pre op AOFAS			Post op AOFAS		
		m1	sd1	n1(feet)	m2	sd2	n2	m1	sd1	n1	m2	sd2	n2	m1	sd1	n1	m2	sd2	n2	m1	sd1	n1	m2	sd2	n2
Endosino tarsal device	Bernasconi A (2019) Orthop Traumatol Surg Res	12	3.1	62	16.8	4.6	62	Nil						18.4	6	62	9.9	3.1	62	nil					
Endosino tarsal device	Megremis P (2019) J Foot Ankle Surg	10.7	2.6	28	15.4	1.1	28	33.8	11.5	28	5.1	3	28	19	6.6	28	0.5	2.7	28	65.1	7.2	28	88.9	5.6	28
Endosino tarsal device	Hsieh CH (2019) JCM	15.3	2	118	16.8	1.3	118	11.1	6.4	118	5.2	0.7	118	12.4	3.2	118	4.8	2.2	118						
Endosino tarsal device group solely	Memeo A (2019) J Foot Ankle Surg (endosinotarsal group)	12.9	1.4	186	16.4	2.3	186	Nil						Nil						Nil					
Endosino tarsal device	Cao L (2017) Orthop Surg	9.4	1.3	27	11.5	1.4	27	19.1	2.2	27	6.3	1.2	27	19.6	1.7	27	4.2	0.9	27	71.1	6.1	27	88.1	6.3	6
Endosino tarsal device	Jay RM (2013) Foot Ankle Spec	Nil						Nil						Nil						67.7	7.9	34	89.0	5	34
Intra-osseous screw	Kubo H (2019) J Orthop Sci	10.6	3.7	95	11.9	3.9	95	Nil						19.1	9.1	95	13.2	6.7	95	Nil					
Exosino tarsal screw group solely	Memeo A (2019) J Foot Ankle Surg (exosinotarsal group)	13	1.5	200	16.6	2.3	200	Nil						Nil						Nil					
Intra-ossoeus screw	Giannini S (2017) J Foot Ankle Surg	Nil						Nil						20.4	7.7	88	9.4	6.5	88	Nil					
Intra-osseous screw	De Pellegrin M (2014) J Child Orthop	11	6	732	14	5	732	Nil						Nil						Nil					
Intra-osseous screw	Jerosch J (2009) Foot Ankle Surg	Nil						Nil						18	8.9	21	6	5.8	21	Nil					
Intra-osseous screw	V. Pavone (2018) J Child Orthop	12.3	2.3	136	16.3	1.3	136	Nil						Nil						79.3	5.7	136	97.3	4.5	136
Intra-osseous screw	Pavone V (2013) J Foot Ankle Surg	12.5	1.4	410	16.7	1.2	410	Nil						Nil						Nil					

### SMD of postoperative radiographic alignment

**Figure 2:**
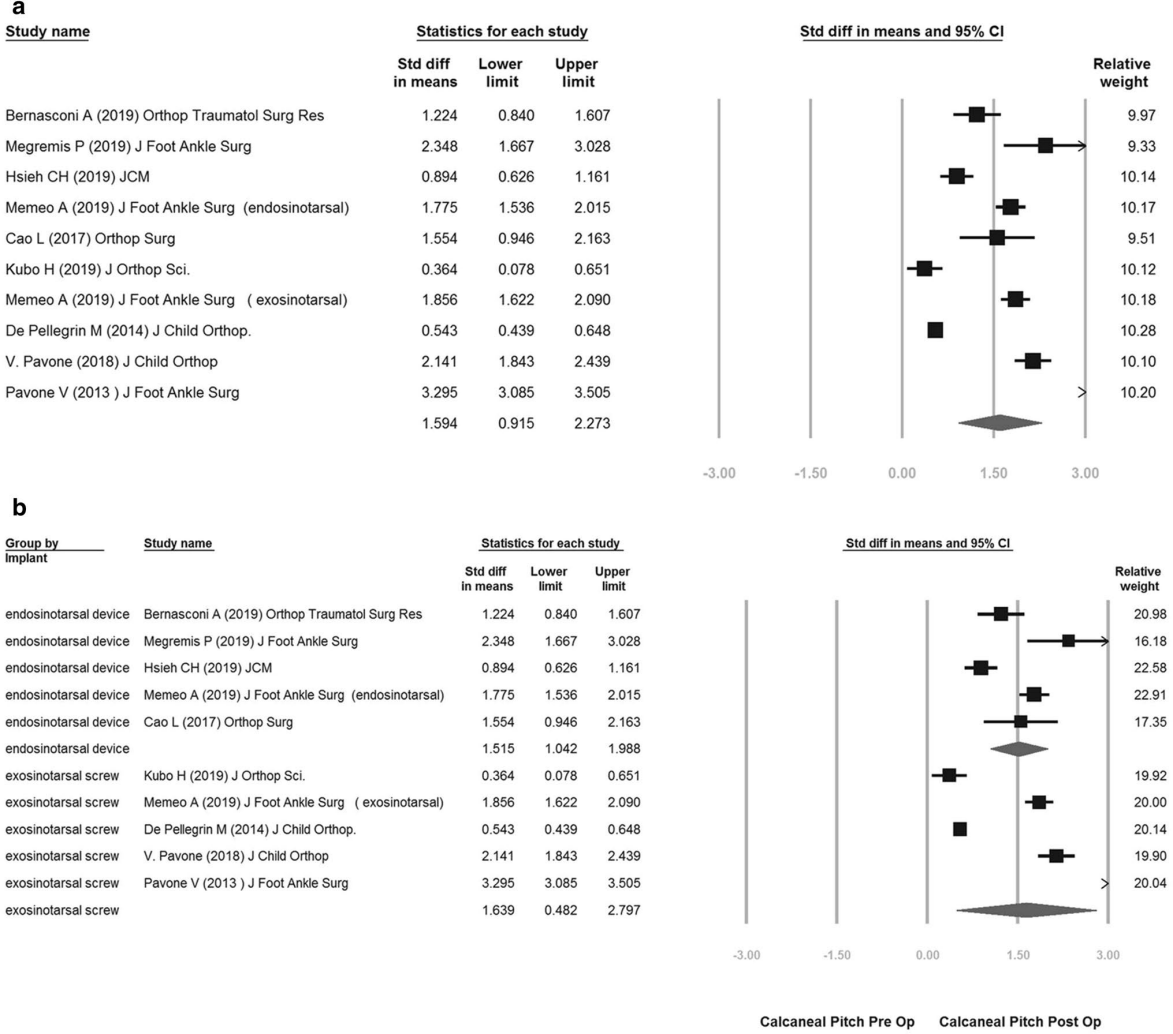
(**a**) overall calcaneal pitch improvement of pre-op vs post-op; (**b**) subgroup improvement of endosinotarsal cone-shaped group vs exosinotarsal screw group.

The overall calcaneal pitch improvement was 1.594 (95% confidence interval [CI] 0.915–2.273) (Fig. 2a).

The subgroup analysis showed both improvement in endosinotarsal cone-shaped group (SMD: 1.515; 95% CI 1.042–1.988) and exosinotarsal screw group (SMD: 1.639; 95% CI 0.482–2.797) (Fig. 2b).

**Figure 3:**
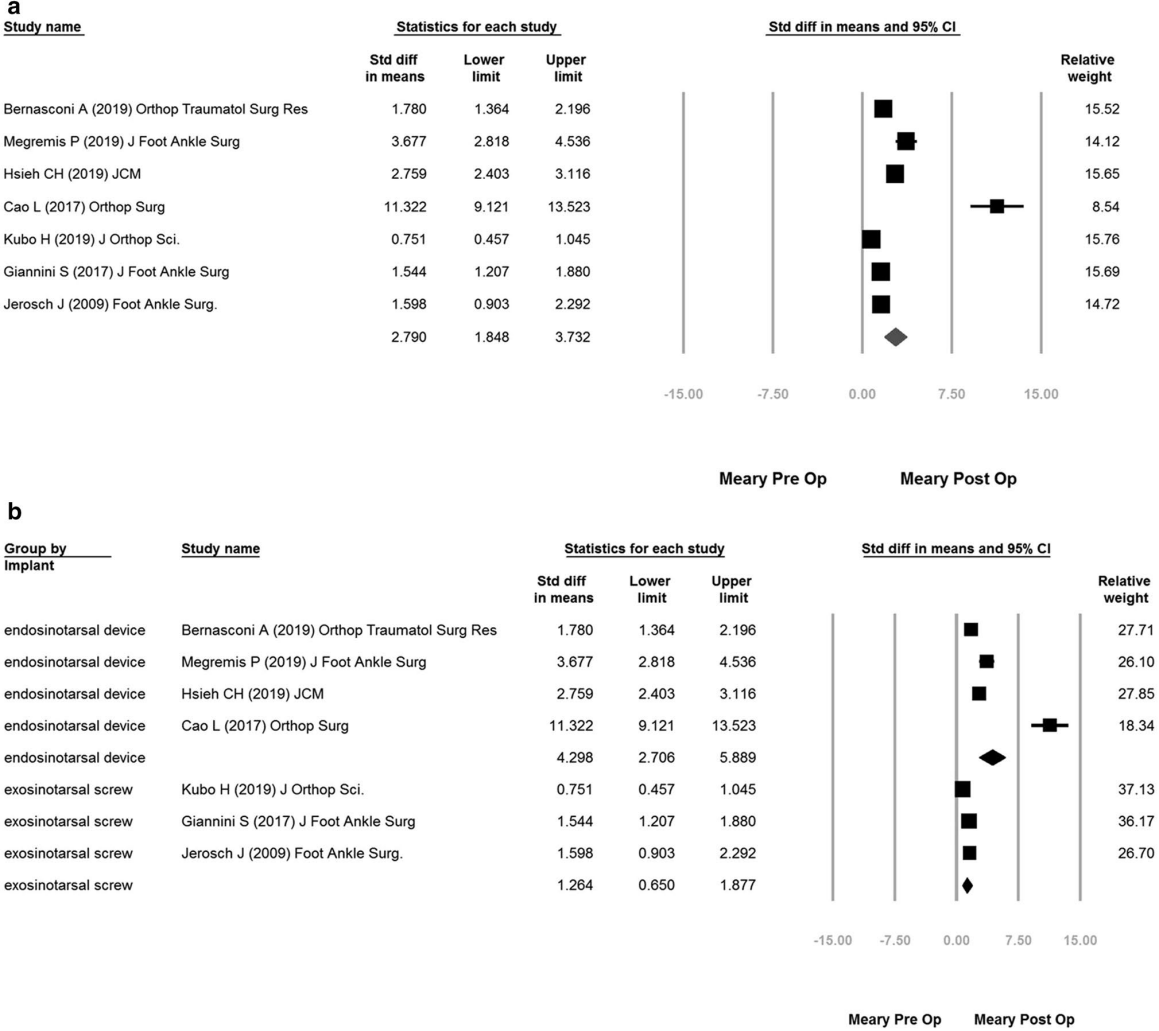
(**a**): overall Meary’s improvement of pre-op vs post-op; (**b**) subgroup improvement of endosinotarsal cone-shaped group vs exosinotarsal screw group.

The overall Meary’s angle improvement was 2.790 (95% confidence interval [CI] 1.848–3.732) (Fig. 3a).

The subgroup analysis showed a more significant improvement in endosinotarsal cone-shaped group (SMD: 4.298; 95% CI 2.706–5.889) than exosinotarsal screw group (SMD: 1.264; 95% CI 0.650–1.877) (Fig. 3b).

### SMD of postoperative clinical outcome score

**Figure 4:**
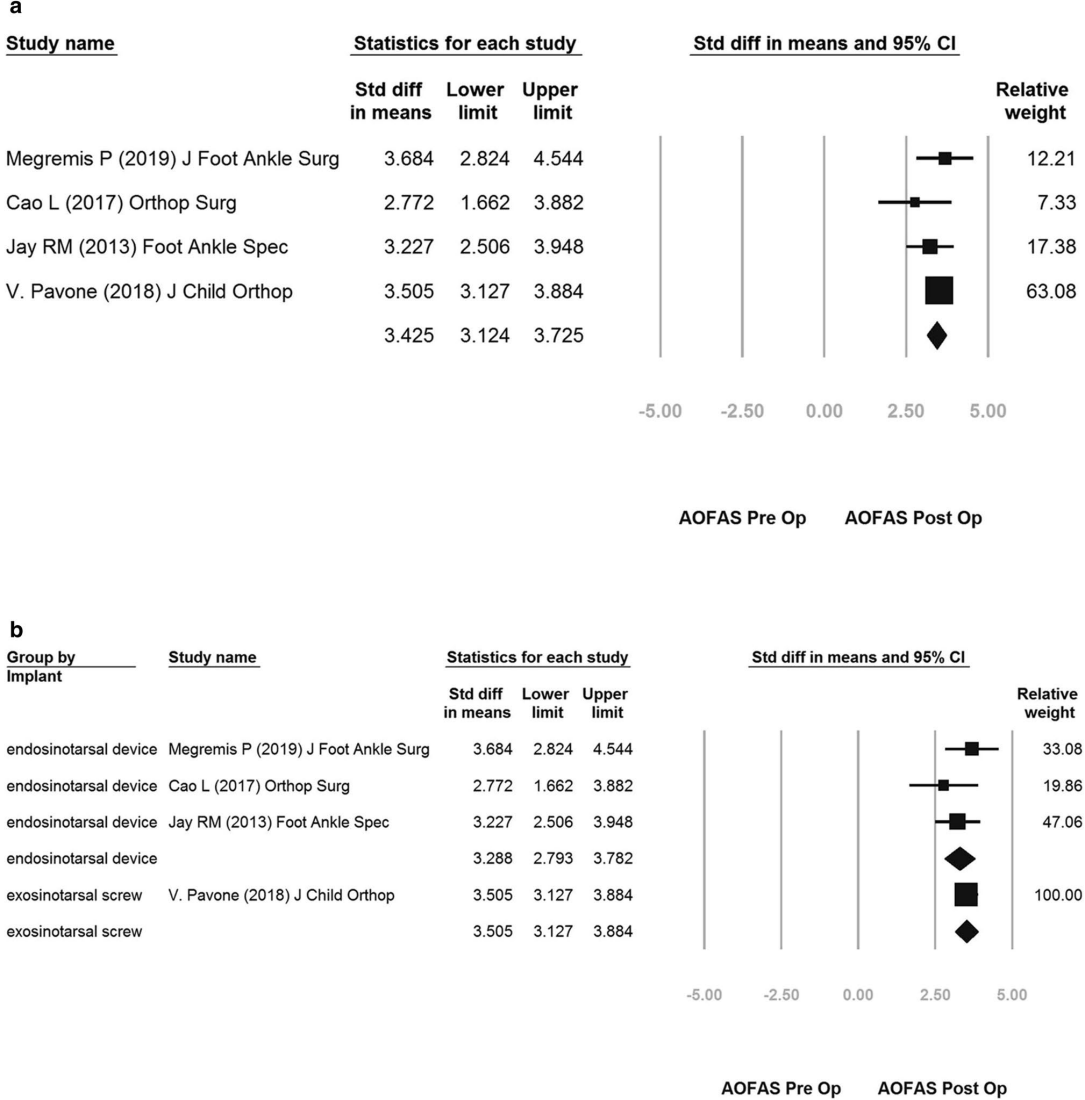
(**a**): overall AOFAS improvement of pre-op vs post-op; (**b**) subgroup improvement of endosinotarsal cone-shaped group vs exosinotarsal screw group.

The overall AOFAS score improvement was 3.425 (95% confidence interval [CI] 3.124–3.725) (Fig. 4a).

The subgroup analysis showed more significant improvement in endosinotarsal cone-shaped group (SMD: 3.288; 95% CI 2.793–3.782) and exosinotarsal screw group (SMD: 3.505; 95% CI 3.127–3.884) (Fig. 4b).

**Figure 5:**
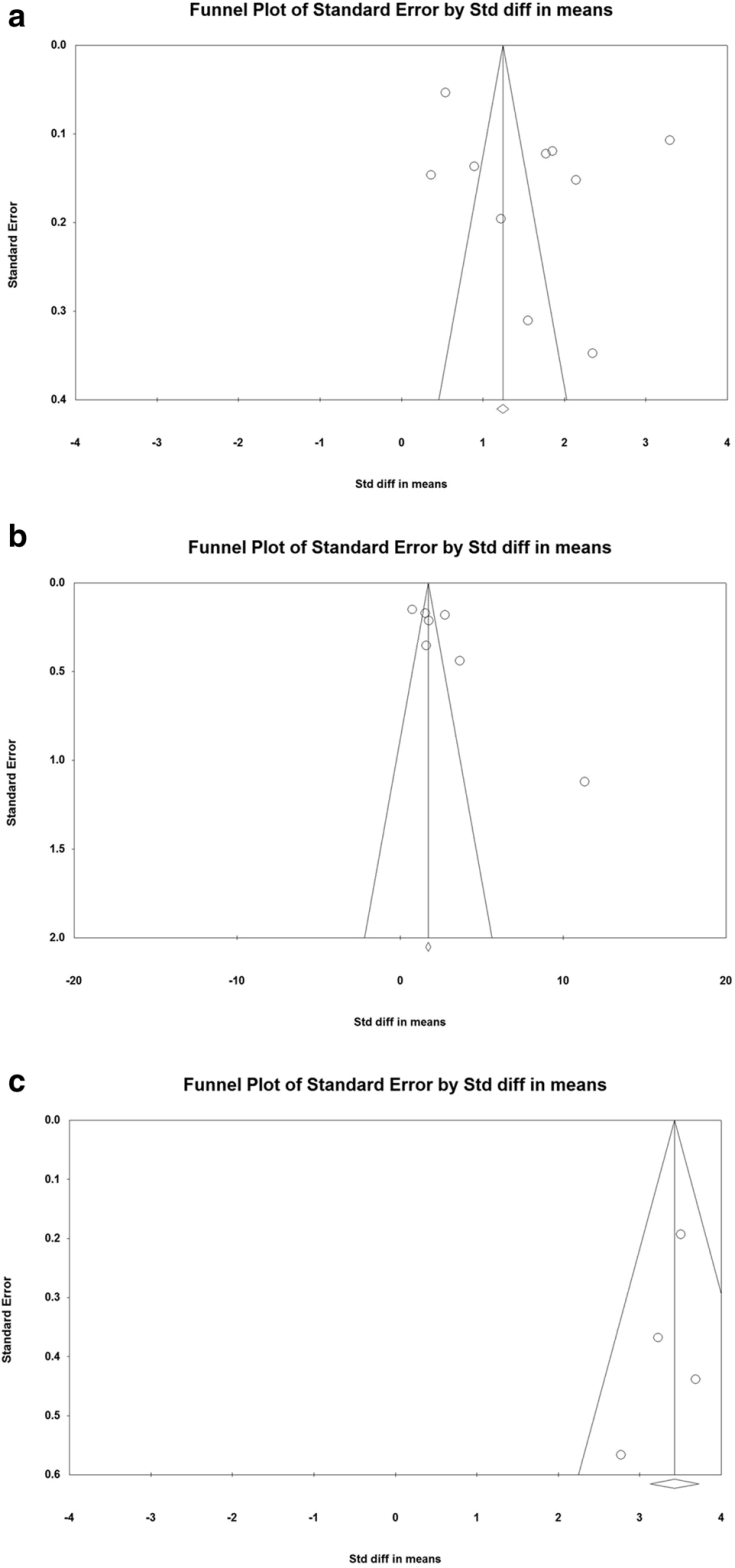
Funnel plots: (**a**) for calcaneal pitch, (**b**) for Meary’s angle, and (**c**) for AOFAS score.

The Egger’s test revealed no significant publication bias regarding the overall calcaneal pitch SMD (*p* = 0.22146), Meary’s SMD (*p* = 0.05507), and AOFAS SMD (*p* = 0.41738). The funnel plot is as shown in Fig. 5a–c for calcaneal pitch, Meary’s angle, and AOFAS score accordingly.

## Discussion

According to Memeo et al., the most common two techniques for subtalar arthroereisis were exosinotarsal metalic screw (SESA) technique and endosinotarsal cone shaped implant. However, previous studies failed to reveal significant difference between the two techniques. Our study thus pooled 12 articles and collected 2063 feet to compare the radiographic and clinical outcomes between the two surgical techniques^1,2^.

Our result revealed both techniques improved in both radiographic parameters. In Meary’s angle, the group of using endosinotarsal cone shaped implant technique improves more than the group using exosinotarsal metallic screw.

Most of previous studies about subtalar arthroereisis did not specifically indicate if the procedure was performed with SESA or endosinotarsal device^2^. This appears that the outcomes in statistics of the studies may be mixed with diverse techniques. In addition, many reviewers could have unclear pictures to recognize which one of the techniques was better for rectifying the pediatric flexible flatfoot. In some articles, authors adopted the same term of “arthroereisis” with different types of implants, even data were from surgeons working in different affiliations. Memeo et al. indicated no statistical difference was found between the two techniques for Costa-Bertani angle, heel inclination, Kite angle and talar declination angle in 402 feet.

Apparently, the data from Memeo A et al. were collected for 10 years with quite complete records. However, with mono-clinical practical surgeries under one hospital, it seems that if we could take a further examination in the event of different hospitals, different surgeons, the result could be shared differently for its consequence.

We assumed the metallic screw had lesser contact area to bone than endosinotarsal implant, which may lead to screw head sinking into bone and thus less corrective effect. The other reason may be due to screw purchasing in cancellous bone with minor fixation effect. Based on above reasons, the results of corrective effect during follow-up did not meet the expectation. Furthermore, the favorite position and screw axis direction performed by surgeons in processing exosinotarsal screw were different. Thus, the screw position was difficult to change after insertion. On the contrary, surgeon can change varieties of endosinotarsal implant in size when the endosinotarsal implant stands at the improper position or appears a flaw of corrective effect. In light of this, the surgeons may change the endosinotarsal implant depending on personal preferences.

Our result revealed both techniques were effective regarding radiographic parameters. For Meary’s angle, the group with endosinotarsal cone shaped implant improved more than the group with exosinotarsal metallic screw. We suggest three possible explanations (there may be more). First, the exosinotarsal metallic screw implant had less contact area to bone than endosinotarsal implants, which may lead to bone erosion and consequent less corrective effect^1,24^. Second, the variable screw purchase to calcaneus may lead to screw loosening after repeated subtalar motion. Last but not least, the optimal position and trajectory for the screw has yet to be determined. Consequently, the stabilizing effect for exosinotarsal screw would be relatively unpredictable. On the contrary, surgeons can modify endosinotarsal implant size according to correcting effects.

There are some factors associated with the occurrence of complications. The type of implant chosen may result in different complication profiles. For example, sinus tarsi pain and implant migration were more frequently seen with endosinotarsal implants. Incomplete correction may be associated with the exosinotarsal implants^30^. Bioabsorbable material may be associated with regional inflammatory response^1,30^. Higher body mass index was found to be associate with higher complication rates and less deformity correction with endosinotarsal implants^21^. Careful selection of patients and techniques based on current evidence may be helpful to reduce the complication rate.

Generally speaking, there are four main types of complications: result of inappropriate indication, technical error, patient adaptation or irritation, and biomechanical failure of implant with incidence of 4.8–18.6%. Complications reported in endosinotarsal device included sinus tarsi pain, implant extrusion, local tissue reaction, and mechanical irritation. Complications reported in extrasinotarsal screw included sinus tarsi pain, subjective limitation, screw breakage, incorrected positioned screw, and local fracture of calcaneus or talus after fall/trauma. While the most common complication is unexpected sinus tarsi pain, the majority of symptoms could be adapted by patient or relieved after implant removal. It seemed to be more easily to handle complications in endosinotarsal device compared with exosinotarsal cases. Up to date, there has been no randomized controlled trial to compare advantages and disadvantages between the two techniques in the current literatures^31–33^.

There are several limitations in our study:

1. Most articles presented these 2 types techniques based on retrospective studies. The quality was limited due to retrospective articles in its nature. Newcastle score is between 5 and 7 with a limited number of extreme high-quality studies.
2. We approached some authors by emails for obtaining more detailed data of standard deviations and implant choices. However, there was no response till the end of our study.
3. The papers related to the outcomes of these two techniques are quite limited. AOFAS is widely used by most investigators. However, it is scattered on QxFAD-C (Oxford ankle foot questionnaire for children). Therefore, there is only one study on AOFAS for Exosinotarsal group.
4. The range of age distribution is relatively wide in both endosinotarsal cone group or exosinotarsal screw group. According to study of Kubo et al.^24^, the best time for the surgeries is 9–12 years old. According to Hsieh et al., most of their patients underwent endosinotarsal implants were before the age of 10, suggesting less bone remodeling in older children. More studies to support the best operation age are required in the future.

The endosinotarsal group had a better corrective result in Meary’s angle compared with exosinotarsal group. For clinical AOFAS, exosinotarsal group also shows an improvement. We assumed that once the sample size was adequate enough, this outcome of clinical AOFAS would be better. Hence, investigators need to examine the outcome of AOFAS clinical scores.

For endosinotarsal device, some articles used bio-absorbable device^1^, while others applied metallic materials. We take conservative attitude to the rectification effectiveness on foot alignment while selecting different materials of endosinotarsal device. Considering the foot alignment needs to sustain a long period under same situation for different materials, age and body weight, future studies will be required for investigating the effectiveness.

Our study provided the evidence of comparing corrective effects of “subtalar arthroereisis” for pediatric flexible flatfoot using different techniques and implants. We suggest that the surgical types, implant selection, sources and costs should be well documented for a more specific statistics in the future.

## Conclusion

The exosinotarsal screw and endosinotarsal device are both effective arthroereisis implants to treat pediatric flexible flatfoot. However, the endosinotarsal device shows a better improvement in Meary’s angle than exosinotarsal screw.

## Data Availability

All the data of the main text are available when *Scientific Reports* requires.
